# The *dmc1* Mutant Allows an Insight Into the DNA Double-Strand Break Repair During Meiosis in Barley (*Hordeum vulgare* L.)

**DOI:** 10.3389/fpls.2019.00761

**Published:** 2019-06-11

**Authors:** Miriam Szurman-Zubrzycka, Brygida Baran, Magdalena Stolarek-Januszkiewicz, Jolanta Kwaśniewska, Iwona Szarejko, Damian Gruszka

**Affiliations:** ^1^Department of Genetics, Faculty of Biology and Environment Protection, The University of Silesia in Katowice, Katowice, Poland; ^2^Department of Plant Anatomy and Cytology, Faculty of Biology and Environment Protection, The University of Silesia in Katowice, Katowice, Poland

**Keywords:** barley, chromosome aberrations, crossing-over, DMC1, meiosis, TILLING

## Abstract

Meiosis is a process of essential importance for sexual reproduction, as it leads to production of gametes. The recombination event (crossing-over) generates genetic variation by introducing new combination of alleles. The first step of crossing-over is introduction of a targeted double-strand break (DSB) in DNA. DMC1 (Disrupted Meiotic cDNA1) is a recombinase that is specific only for cells undergoing meiosis and takes part in repair of such DSBs by searching and invading homologous sequences that are subsequently used as a template for the repair process. Although role of the *DMC1* gene has been validated in *Arabidopsis thaliana*, a functional analysis of its homolog in barley, a crop species of significant importance in agriculture, has never been performed. Here, we describe the identification of barley mutants carrying substitutions in the *HvDMC1* gene. We performed mutational screening using TILLING (Targeting Induced Local Lesions IN Genomes) strategy and the barley TILLING population, *Hor*TILLUS, developed after double-treatment of spring barley cultivar ‘Sebastian’ with sodium azide and *N*-methyl-*N*-nitrosourea. One of the identified alleles, *dmc1.c*, was found independently in two different M_2_ plants. The G2571A mutation identified in this allele leads to a substitution of the highly conserved amino acid (arginine-183 to lysine) in the DMC1 protein sequence. Two mutant lines carrying the same *dmc1.c* allele show similar disturbances during meiosis. The chromosomal aberrations included anaphase bridges and chromosome fragments in anaphase/telophase I and anaphase/telophase II, as well as micronuclei in tetrads. Moreover, atypical tetrads containing three or five cells were observed. A highly increased frequency of all chromosome aberrations during meiosis have been observed in the *dmc1.c* mutants compared to parental variety. The results indicated that DMC1 is required for the DSB repair, crossing-over and proper chromosome disjunction during meiosis in barley.

## Introduction

Meiosis is a process of essential significance for sexual reproduction. During meiosis distribution of genetic material to gametes is associated with recombination which is achieved through crossing over and chromosome segregation. The recombination event (crossing-over) takes place during the first meiotic prophase between non-sister chromatids of homologous chromosomes. It leads to establishing physical links between homologous chromosomes, called chiasmata. Meiotic crossing-over shuffles genetic information, creates new combinations of alleles, and therefore generates genetic variations and drives evolution. The process of recombination during meiosis starts with a programmed DNA double-strand break (DSB). Meiotic DSBs are introduced through the catalytic action of the evolutionarily-conserved SPO11 (Sporulation Protein 11) protein complex which is an enzyme related to type II DNA topoisomerases (Keeney et al., 1997; Robert et al., 2016; Vrielynck et al., 2016). In general, DSBs can be repaired through two major pathways: homologous recombination (HR) and/or non-homologous end joining (NHEJ) (Ohnishi et al., 2009). Programmed DSBs during meiosis are eliminated by HR in the DSBR pathway (Double Strand Break Repair model). The model of DSB repair was first proposed by Szostak and coworkers in the 1980s (Szostak et al., 1983). The extensive studies of this process in *Saccharomyces cerevisiae* implemented only several alterations to the original model (reviewed in Andersen and Sekelsky, 2010). After introduction of DSB, the DNA ends are resected and long (about 1 kbp) 3′single-stranded overhangs are created, called 3′ssDNA tails (Ohnishi et al., 2009). RAD51 (Radiation sensitive 51) and DMC1 (Disrupted Meiotic cDNA1) recombinases attach to these tails and form nucleoprotein filaments that search for and invade homologous sequences either on a sister chromatid or on a homologous chromosome (Bishop et al., 1992; Shinohara et al., 1992). The latter case may lead to genetic recombination. After invasion on homologous sequence, the next step in the DSBR pathway is establishing the D-loop structure followed by formation of a double Holliday Junction (dHJ) intermediate. Then, the two strands at each HJ are nicked by specific enzymes and ligated. The resolution of dHJ can result in both, crossover and non-crossover repair products (COs and NCOs, respectively) (Andersen and Sekelsky, 2010). DMC1 and RAD51 belong to the same protein family of recombinases, involved in DNA repair through HR, which are related to the bacterial RecA (Bianco et al., 1998). They catalyze the process of pairing and invasion of 3′ssDNA tails formed at the DSB sites into homologous double-stranded DNA. Both of these proteins take part in the meiotic recombination events, however, DMC1 is specific only for cells undergoing meiosis, while RAD51 is ubiquitous and acts also in DSB repair in somatic cells. It is suggested that DMC1 promotes only the CO recombination with the homologous chromosome, which is unique to meiosis, and RAD51 plays its role mainly in sister chromatid exchange or the NCO recombination (Shinohara and Shinohara, 2004; Neale and Keeney, 2006). However, a recent work has shown that in the case of absence of the RAD51-mediated strand exchange activity, the DMC1 activity is sufficient to repair all DSBs during meiosis into both CO and NCO products and it does not affect meiotic crossing-over rates or patterns (Cloud et al., 2012; Da Ines et al., 2013; Singh et al., 2017).

In the plant kingdom, meiosis has been studied to the greatest degree in *Arabidopsis thaliana* (for review see Mercier et al., 2015). Cereals with large genomes and large chromosomes, such as barley (*Hordeum vulgare*), are characterized by highly skewed distribution of meiotic crossovers. Consequently, the large sub-centromeric regions, representing substantial proportions of the physical map, are seldom recombined (Higgins et al., 2012; Ramsay et al., 2014). Therefore, the molecular mechanisms underlying meiotic events may be distinct for model Arabidopsis with genome size of ∼135 Mbp (The Arabidopsis Genome Initiative [AGI], 2000) contained within five chromosomes and for barley with genome size of ∼5.3 Gbp contained within seven chromosomes (International Barley Genome Sequencing Consortium Mayer et al., 2012; Mascher et al., 2017). Our knowledge on the DMC1 function in plants comes mainly from studies performed in Arabidopsis. Moreover, its detailed function in DSB repair during meiosis is still extensively discussed. For example, some contradictory reports have appeared in rice (*Oryza sativa* L.): one, showing that OsDMC1 is required for homologous pairing (Deng and Wang, 2007), and the other, reporting that it is dispensable in this process (Wang et al., 2016), which is different from the role of DMC1 described in other species. These results imply that the function of DMC1 may be distinct in diverse organisms and a direct transfer of knowledge from related species may not be feasible. The recent findings in rice have been obtained studying rice insertion mutants (Wang et al., 2016). Although some *in silico* studies of DMC1 have been performed in monocot crops, including barley (Barakate et al., 2014), only very recently role of the barley homolog was analyzed in a spontaneous mutant (Colas et al., 2019).

Barley (*Hordeum vulgare* L.), ranking fourth in production and acreage, belongs to the most important cereal crops worldwide. Here, we present the identification of barley mutants in the *DMC1* gene isolated using TILLING strategy in the *Hor*TILLUS population derived from chemical mutagenesis of spring cultivar ‘Sebastian’. Cytological analysis of male meiocytes in the identified *dmc1* mutants revealed various abnormalities during meiosis, in anaphase/telophase I and anaphase/telophase II, as well in tetrads. Our results indicate that DMC1 is involved in the DSB repair, crossing-over and chromosome disjunction during meiosis process in barley.

## Materials and Methods

### Plant Material

The *Hor*TILLUS (*Hordeum vulgare* – TILLING – University of Silesia) population has been used for mutation detection in the *HvDMC1* gene through TILLING approach. This population was developed after double treatment of spring barley cultivar ‘Sebastian’ with sodium azide and *N*-methyl-*N*-nitrosourea (Szurman-Zubrzycka et al., 2018). Each M_2_ plant of the *Hor*TILLUS population originated from a different M_1_ plant. Eight-fold DNA pools from M_2_ *Hor*TILLUS plants served as templates for mutational screening. The homozygous lines of the isolated *dmc1* mutants were backcrossed with their parent variety and homozygous mutants selected from the F_2_ populations have been used for cytological analyses of meiosis. Barley cv. ‘Sebastian’ has been used as a wild type in this study.

### Mutational Screening in *HvDMC1* Using the TILLING Strategy

**FIGURE 1:**
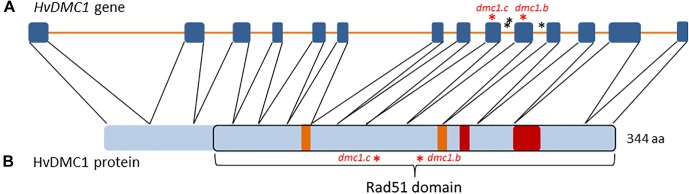
**(A)** The structure of the *HvDMC1* gene. Blue boxes symbolize exons and orange lines introns. The identified mutations are indicated by asterisks (red – missense mutations, black – mutations in non-coding regions). **(B)** The structure of the HvDMC1 protein with localization of the Rad51 domain. Orange boxes symbolize the Walker A and Walker B functional motifs that are responsible for ATP binding, red boxes symbolize ssDNA-binding loops 1 and 2. The positions of amino-acid residues mutated in the *dmc1.c* and *dmc1.b* alleles are indicated by red asterisks.

**FIGURE 2:**
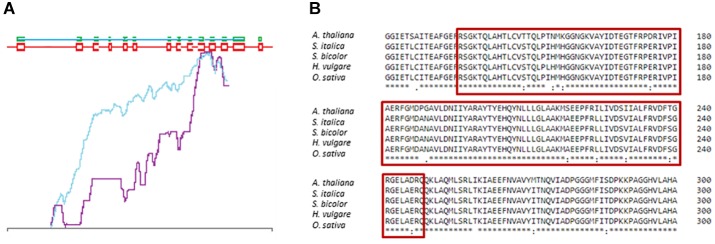
Selection of the *HvDMC1* gene fragment for TILLING analysis **(A)** CODDLE analysis showing the most conserved fragment of the gene (blue line – score missense changes and truncations; violet – the PSSM difference). **(B)** Comparison of the DMC1 protein sequences from various plant species. The red frame shows conserved fragment of the protein which is encoded by the analyzed fragment of the *HvDMC1* gene.

The sequence of the *DMC1* gene in barley was identified and published by Klimyuk et al. (2000) in the NCBI database (Acc. no. AF234170.1). Its genomic and coding sequences consist of 5654 bp and 1035 bp, respectively. The *HvDMC1* gene is composed of 14 exons and encodes a protein which is 344 amino acid in length (Figure 1). Our bioinformatics analysis revealed that *HvDMC1* gene has no paralogs in barley genome (Supplementary Materials 1, 2). The DMC1 sequence is strongly conserved among various species representing the plant and animal kingdom. The bioinformatics tools: ClustalOmega^1^ and CODDLE (Codons Optimized to Discover Deleterious Lesions) were used to select fragment of the *HvDMC1* gene for mutational screening. This *in silico* analysis enabled selection of the gene fragment which is highly conserved among homologous sequences from different plant species (Figure 2). Sequence encoding the Rad51 functional domain, which is characteristic for proteins involved in the DNA repair, was mapped in the *HvDMC1* gene with the use of Pfam tool^2^. Based on these bioinformatics analyses, the 811 bp long fragment of the *HvDMC1* gene containing exons 7 to 11, encoding a part of the Rad51 domain, was chosen as an amplicon for the TILLING screening. PCR reaction was optimized for specific primers labeled with IRDye-700 (forward) and IRDye-800 (reverse) (Supplementary Material 3). TILLING was performed on DNA of 5,376 M_2_ plants of the *Hor*TILLUS population. The method of mutational screening applied in this study was performed according to the protocol described elsewhere (Szurman-Zubrzycka et al., 2017; Jost et al., 2019). Briefly, the eight-fold pools were used for PCR reaction with IRDye-700 and IRDye-800 labeled and unlabeled primers (Supplementary Material 3). The next step, formation of heteroduplexes, was performed at 95°C for 3 min for initial denaturation, and then at 70°C for 20 sec (×70 cycles, -0.1°C per cycle) for slow renaturation. Heteroduplexes appeared only in pools with mutations within the analyzed amplicon. After heteroduplex formation the samples were treated with 20 μl of 0.1× Celery Juice Extract (CJE) containing Cel I enzyme that specifically recognizes and cuts DNA mismatches. The enzymatic cleavage was performed at 45°C for 15 min. The products of cleavage were purified with 96% ethanol with 1% sodium acetate and then washed with 70% ethanol. After centrifugation the pellets were dried and dissolved in 3 μl of STOP buffer (containing 5% bromophenol blue-xylene, 40% formamide and 1% EDTA). Before loading on polyacrylamide gel the samples were denatured. The electrophoresis was carried out in LI-COR sequencers in denaturing 6% polyacrylamide gels in 1xTBE (Tris – Boric Acid – EDTA) running buffer at the following settings: 3000 V, 30 mA and 30 W. The lanes with additional bands indicating putative mutations in the analyzed bulks were selected for further analysis (Supplementary Material 4). For identification of single plants carrying the mutations, each sample from the selected bulk was then analyzed by mixing its DNA individually with DNA of the parent variety following the same method described for the eight-fold pools. The analyzed fragments from the identified plants were sequenced in order to confirm the presence of mutations.

### Preparation of Material for Microscopic Analyses of Meiosis

Plants of the *dmc1* mutants as well as their parental cultivar ‘Sebastian’ were grown in a greenhouse at 22/20°C (day/night, respectively), under a photoperiod of 16 h/8 h and a light intensity of 400 μE/m^2^s for approximately 4 months, until their spikes reached length of 2.5–5 cm. Immature ears were harvested and immediately fixed in the methanol:acetic acid (3:1, v/v) overnight at room temperature.

To investigate the involvement of HvDMC1 in the DSB repair during meiosis, anthers from immature florets were used for preparation of male meiocyte spreads. Only cells in meiotic phases after crossing over were analyzed. Particular focus was given on cells in anaphase I/II and telophase I/II as well as tetrads, in which it was possible to observe micronuclei.

Cytogenetic slides were prepared using the Feulgen’s squash technique. Three anthers were isolated to prepare one slide. Cytological analyses were performed for each genotype in three repetitions with 15 slides per replica. The frequencies of anaphase/telophase I and anaphase/telophase II cells with chromosome aberrations were analyzed, on average, in 158 and 165 cells per slide, respectively. The frequencies of cells in tetrad stage with the micronuclei were estimated, based on analysis of, on average, 170 cells per slide. Preparations were examined with the Nikon ECLIPSE Ni bright field microscope. Images were captured by the Nikon DS.- Fi1c camera under 40× magnification.

## Results

### Mutation Identification and Characterization

After screening of 5,376 M_2_ plants of the *Hor*TILLUS population, six independent mutations in the *HvDMC1* gene were identified (Table 1). All identified mutations were confirmed by sequencing and all of them are G/C to A/T transitions. Based on the number of the identified mutations in the *HvDMC1* gene (6), the length of amplicon (811 bp) and the number of M_2_ plants screened (5,376), the calculated mutation density in this gene was 1 mutation per 729 kbp.

**Table 1:** Characteristics of mutations identified in the *HvDMC1* gene.

Allele	No of M_2_ plant	Mutation position in genomic sequence	Type of mutation	Alteration in protein sequence	SIFT score	Mutation state in M_2_ plant
*dmc1.a*	1519/001	C2515T	NC	–		Homozygous
*dmc1.b*	1694/001	G2742A	MS (exon 10)	G212S	0,43	Homozygous
*dmc1.c*	3041/001	G2571A	MS (exon 9)	R183K	0,03	Heterozygous
*dmc1.c*	3223/001	G2571A	MS (exon 9)	R183K	0,03	Heterozygous
*dmc1.d*	3896/002	C2495T	NC	–		Homozygous
*dmc1.e*	3976/001	C2837T	NC	–		Heterozygous


*NC, mutation in non-coding sequence; MS, missense mutation.*

**FIGURE 3:**
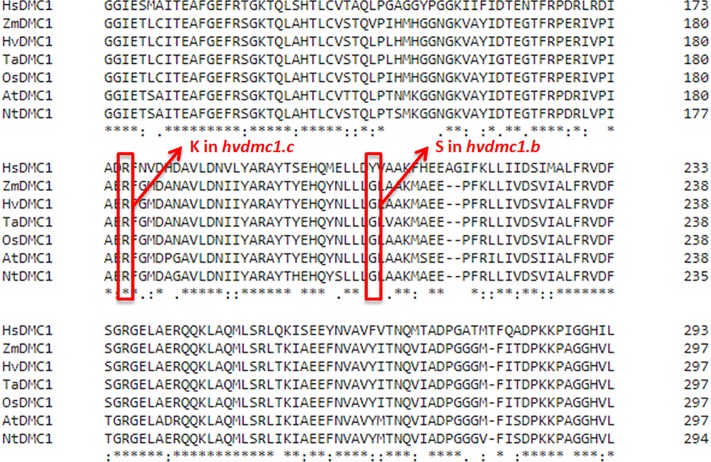
Multiple alignment of fragment of the DMC1 protein sequences from various species with positions of substituted amino acids in the *dmc1.b* and *dmc1.c* mutants indicated with red frames. *Hs – Homo sapiens, Zm – Zea mays, Hv – Hordeum vulgare, Ta – Triticum aestivum, Os – Oryza sativa, At – Arabidopsis thaliana, Nt – Nicotiana tabacum*.

Six mutations identified in the *HvDMC1* gene gave five new alleles (*dmc1.a* – *dmc1.e*) (Table 1 and Figure 1). The same mutation G2571A (the *dmc1.c* allele) was induced and identified independently in two different M_2_ plants which originated from different M_1_ individuals: plant no. 3041/001 and plant no. 3223/001. To distinguish the origin of the mutated allele, it is hereafter named as *dmc1-3041* or *dmc1-3223* depending on the mutated line. Three mutations – *dmc1.a*, *dmc1.d* and *dmc1.e* occurred in non-coding, intron regions of the *HvDMC1* gene. They were analyzed *in silico* and the positions of these mutations are neither in donor/acceptor sites of introns nor in polypyrimidine tracts or branch points, so they are probably not essential for splicing and do not have any impact on the encoded protein. Homozygous plants carrying these intronic mutations did not show any visible morphological changes when compared to ‘Sebastian’. Three other mutations – *dmc1.b*, *dmc1.c-3041* and *dmc1.c-3223*, occurred in coding sequence (*dmc1.b* in exon 10, *dmc1.c* in exon 9) and they cause amino acids alterations at the protein level. The *dmc1.b* mutation changes glycine-212 to serine (G212S) and the *dmc1.c* mutation changes arginine-183 to lysine (R183K). Potentially, both of them can be used for functional analysis of the *DMC1* gene in barley. The SIFT (Sorting Intolerant From Tolerant) tool was used to analyze *in silico* the influence of the identified mutations on protein activity and functioning. If the SIFT score is less than 0.05 the mutation is considered as deleterious for protein activity (Ng and Henikoff, 2003; Kumar et al., 2009). According to this bioinformatics analysis, the *dmc1.b* mutation is functionally neutral (SIFT score = 0.43), whereas the *dmc1.c* mutation is deleterious (SIFT score = 0.03). The multiple alignment of the DMC1 proteins from various species showed that the amino acid substituted in the *dmc1.b* mutant (glycine-212) is conserved among plant species, while the amino acid changed in the *dmc1.c* mutant (arginine-183) is conserved not only among plants, but also in *Homo sapiens* (Figure 3). This also suggests that the *dmc1.c* mutation may have more significant impact on the protein function, nevertheless for further investigation we have used all three mutant lines carrying missense mutations – *dmc1.b*, *dmc1.c-3041* and *dmc1.c-3223*. We have developed homozygous mutant lines and used plants of the M_4_/M_5_ generation for backcross with their parent variety ‘Sebastian’ in order to reduce the number of putative background mutations. We selected homozygous mutant plants form the BC (backcross) F_2_ generations and used them for cytological analysis of meiosis.

### Cytological Observations of Meiosis in the *hvdmc1.b*, *hvdmc1.c-3041* and *hvdmc1.c-3223* Mutants

**FIGURE 4:**
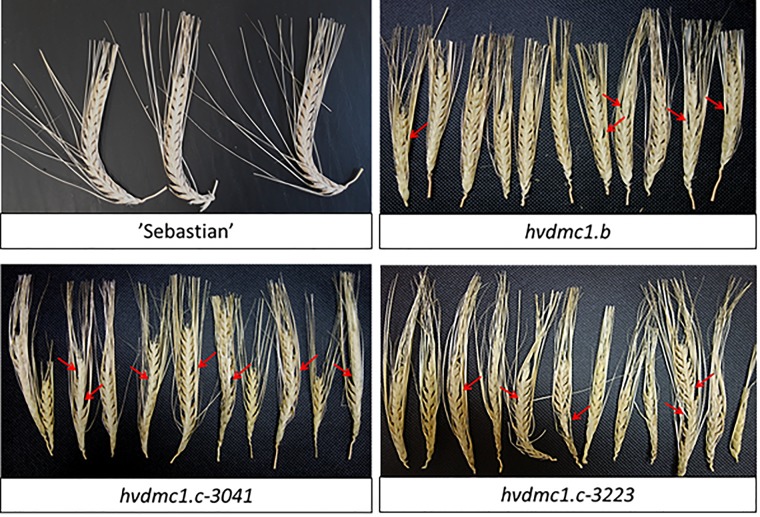
Spikes of cv. ‘Sebastian’ and the *dmc1.b*, *dmc1.c-3041*, and *dmc1.c-3223* mutants displaying partial sterility. Red arrows indicate examples of places where no grains were developed.

**FIGURE 5:**
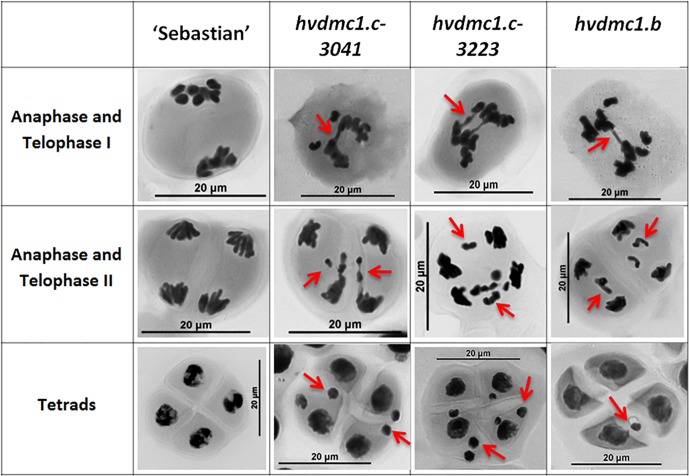
The summary panel with the examples of meiotic cells in different stages: anaphase/telophase I, anaphase/telophase II and in tetrads in ‘Sebastian’ and the *dmc1.c-3041, dmc1.c-3223 and dmc1.b* mutants. In the anaphase and telophase I the chromosome bridges, in the anaphase and telophase II the chromosome fragments, whereas in tetrad stage the micronuclei are indicated by red arrows.

**FIGURE 6:**
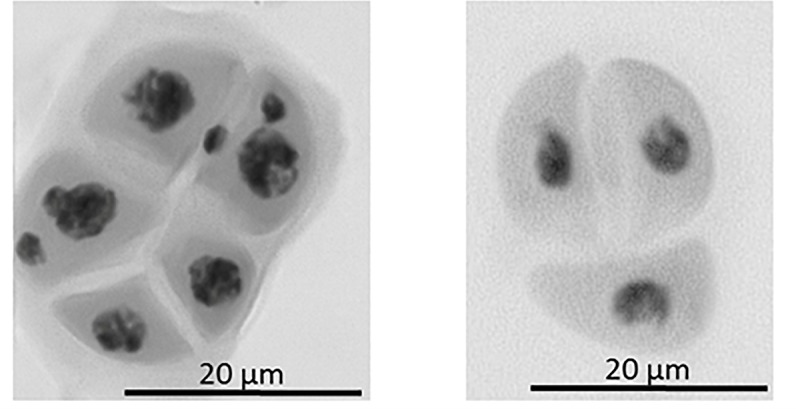
Example of abnormal tetrad formation in the *dmc1.c-3041* mutant. On the left: tetrad composed of five cells, two of the cells show the presence of two micronuclei. On the right: tetrad composed of three cells, no micronuclei are present.

**FIGURE 7:**
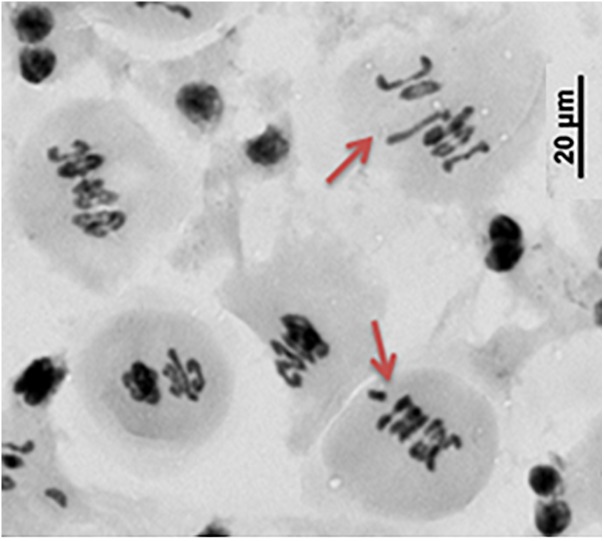
The abnormal chromosome assembling during metaphase I in the *dmc1.c-3223* mutant.

The first observed phenotypic feature of all the identified *dmc1* mutants selected for the cytological analysis was partial sterility of their spikes, which indicated some fertility disorders (Figure 4). Apart from that, the mutants did not show any evident morphological changes when compared to the wild type ‘Sebastian’. In all three *dmc1* mutants we observed chromosome aberrations such as chromosomal bridges and chromosome fragments during anaphase and telophase I and II and micronuclei in tetrads (Figure 5). In the *dmc1.c-3041* mutant an abnormal tetrads consisting of three or five haploid cells were observed (Figure 6). Additionally, we observed that both *dmc1.c* mutants showed disturbances in the formation of bivalents plate in metaphase I (Figure 7). However, this observation needs further investigations.

**FIGURE 8:**
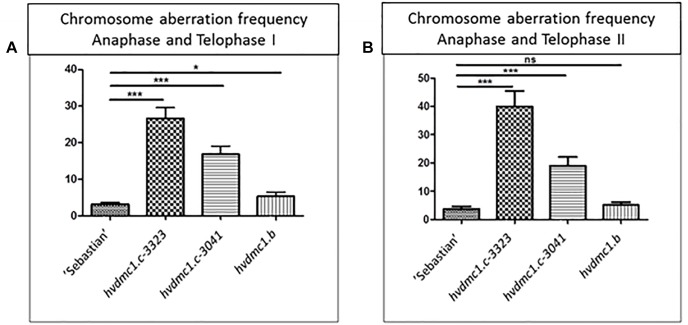
**(A)** Comparison of the chromosome aberration frequencies in anaphase and telophase I in ‘Sebastian’ (wt) and three *dmc1* mutants. **(B)** Comparison of the chromosome aberration frequencies in anaphase and telophase II in ‘Sebastian’ and three *dmc1* mutants. Stars indicate statistical significant differences (ANOVA; *p* < 0,05) between ‘Sebastian’ and the three *dmc1* mutants, ns – differences between compared genotypes were not statistically significant.

**FIGURE 9:**
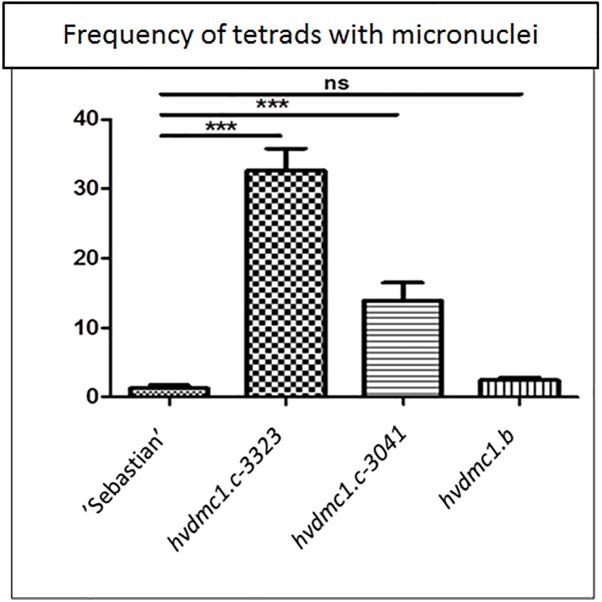
Comparison of the frequency of tetrads with micronuclei in ‘Sebastian’ (wt) and three *dmc1* mutants. Stars indicate statistically significant differences (ANOVA; *p* < 0,05) between ‘Sebastian’ and the three *dmc1* mutants, ns – differences between compared genotypes were not statistically significant.

In all analyzed meiosis phases, the *dmc1.c-3041* and *dmc1.c-3223* mutants displayed statistically significant differences in chromosomal aberration frequency compared to the wild type variety ‘Sebastian’ (Figure 8, 9). In anaphase/telophase I, the parent variety exhibited the chromosome aberration frequency at the level of 3%, whereas in the *dmc1.c* mutants the chromosome aberration frequency was significantly higher: 17% and 26% in *dmc1.c-3041* and *dmc1.c-3223*, respectively (Figure 8A). In anaphase/telophase II, the frequency of cells with chromosome aberrations in the mutants was 19 and 40% (in the *dmc1.c-3041* and *dmc1.c-3223*, respectively; Figure 8B) compared to 4% in ‘Sebastian’. The frequency of tetrads with micronuclei was also significantly higher in the mutants – 14 and 33% (in *dmc1.c-3041* and *dmc1.c-3223*, respectively) than in the wild type, where it reached 1% (Figure 9).

The *dmc1.b* mutant also showed abnormalities during meiosis, but with much lower frequencies than the *dmc1.c* mutants. In anaphase and telophase I, the frequency of chromosomal aberrations in the *dmc1.b* mutant was 5.5%, about two times higher than in the wild type ‘Sebastian’. In anaphase and telophase II, the frequency of chromosome aberration (5%) observed in *dmc1.b* did not differ statistically from the parent variety ‘Sebastian’ (4%) (Figure 8). Analysis of micronuclei in tetrads has also shown no significant differences between this mutant and its parent variety (Figure 9).

## Discussion

We have performed the analysis of meiosis in the *dmc1.b* mutant carrying missense mutation leading to the G212S substitution and both the *dmc1.c* lines (*dmc1.c-3041* and *dmc1.c-3223*) carrying missense mutation causing the R183K change. Observation of meiosis in the mutants revealed that the *dmc1.c* lines showed differences in chromosomal aberrations frequency when compared to the wild type, whereas the *dmc1.b* mutant did not show significant disorders. This results are consistent with the *in silico* analysis of conservation of the substituted amino acid positions and with the SIFT values, which have shown that the *dmc1.c* mutation should have a more significant impact on protein function than *dmc1.b*.

Our *dmc1.c* mutants exhibited very high chromosome aberration frequencies in anaphase/telophase I/II and also very high number of tetrads with micronuclei. We suggest that these changes are the result of defects in DSB repair and anomaly in crossing-over, what strongly confirms that the *DMC1* gene is involved in the DSB repair, recombination and chromosome disjunction during meiosis. Both *dmc1.c* mutants showed disturbances in the chromosome assembling during the metaphase I. Such abnormalities have previously been observed in other species, both in plants and animals. The *DMC1*-knock-out mice displayed aberrant chromosomal pairing or non-homologous chromosome pairing in spermatocytes (Habu et al., 1996). Our findings are also consistent with the observations of the *atdmc1* mutants which exhibited abnormalities in the formation of bivalents and chiasmata (Da Ines et al., 2013). Moreover, in meiotic cells of the rice *osdmc1a osdmc1b* insertional double mutant, univalents and abnormal number of chromosomes in the metaphase plate during the second meiotic division were observed (Wang et al., 2016). In our study, one mutated line, *dmc1.c-3041*, formed irregular tetrads containing three or five haploid cells. Similar anomalies were observed in the rice *osdmc1a osdmc1b* double mutant (Wang et al., 2016). This type of anomaly may be the result of abnormal, uneven segregation of chromosomes to the opposite poles of the cell during meiotic divisions. The *dmc1.b* as well as both *dmc1.c* mutant lines show partial sterility of the spikes. Previously reported *dmc1* mutants in different species showed sterility or partial sterility. For example, rice *OsDMC1*-RNAi lines, as well as the insertional *osdmc1a osdmc1b* double mutant grow normally during their vegetative phase, but they are characterized by total sterility (Deng and Wang, 2007; Wang et al., 2016). The insertional Arabidopsis mutant, *atdmc1*, produces viable seeds at very low ratio (1.5%) (Da Ines et al., 2013). The *DMC1* knock-out mice displayed total sterility (Habu et al., 1996). This suggests that fertility disorders are a common feature of individuals lacking the *DMC1* gene in different species. Our *dmc1* mutants are not a knock-out type, they carry missense mutations in the analyzed gene and, as it was predicted in our analysis (Figure 1), the substituted amino-acid residues are located in the Rad51 domain, however outside the functional motifs of the DMC1 protein (such as the Walker A and B motifs, and loops 1 and 2), therefore the effect on sterility is not that strong as in other species.

In our study the mutational screening of 5,376 M_2_ plants from the *Hor*TILLUS population revealed six independent G/C to A/T mutations within the *HvDMC1* gene. Most mutations (88%) found to date in the *Hor*TILLUS population represented this type of transition (calculated based on data of 32 genes TILLed; Szurman-Zubrzycka et al., 2018). Both mutagens used for creation of our TILLING population (MNU and NaN_3_) cause such DNA lesions. *N*-methyl-*N*-nitrosourea belongs to alkylating agents that are known to alkylate guanine and create O^6^-metG – the lesion with strong mutagenic property (Kleibl, 2002). O^6^-metG mispairs with thymine, which leads to its replacement by adenine in the subsequent replication cycle. If this methylation is induced in a non-transcribed (sense) DNA strand, it leads to G to A transition, whereas if it occurs in transcribed (antisense) DNA strand it results in C to T transition. TILLING populations which were developed after treatment with MNU for *Glycine max* and *Oryza sativa* showed 89.4 and 91.7% G/C to A/T transitions, respectively (Cooper et al., 2008; Suzuki et al., 2008). Sodium azide, the other mutagen used for establishing the *Hor*TILLUS population, is mutagenic only for some plant species, among them barley and rice (reviewed in Gruszka et al., 2012). It was used as the only mutagen in other barley TILLING population - TILLMore developed for cultivar ‘Morex’, where it caused mainly G/C to A/T transitions (95.5%, Talamè et al., 2008; Sparla et al., 2014). The mutation density calculated based on mutations found in the *HvDMC1* amplicon is 1 per 729 kbp. The average mutation density in the *Hor*TILLUS population is 1 per 477 kbp, however it varies between gene fragments (Szurman-Zubrzycka et al., 2018). The value obtained for *HvDMC1* is slightly lower, what could be caused by amplicon base content (G/C – 42%; A/T – 58%).

One very important aspect in terms of functional genetics is the presence of paralogs within the genome that can take over functions of gene of interest (functional redundancy). If there are two or more closely related genes, usually it is necessary to produce individuals with mutations in both or all of paralogs to perform functional analysis. Barley genome is one of the largest diploid genomes sequenced with a haploid genome size of more than 5 Gbp in seven large chromosomes (International Barley Genome Sequencing Consortium Mayer et al., 2012; Mascher et al., 2017). In order to check if there are any paralogs of *HvDMC1* in barley genome we have screened its 2nd version that has been recently released (Mascher et al., 2017) with the use of the EnsemblPlants^3^ and the IPK Barley BLAST^4^ servers. Our analysis indicated that the *HvDMC1* gene (HORVU5Hr1G040730) is located on chromosome 5 and has no paralogs in the genome (Supplementary Materials 1, 2), so our *dmc1.b* and *dmc1.c* mutants are good tools to study the function of this gene, because the risk of gene redundancy is very low.

An issue which is sometimes raised considering TILLING mutants is that observed phenotype may be caused by other, than analyzed, mutations in the genome (so called background mutations). Taking into consideration the size of barley genome and the overall mutation density found in the *Hor*TILLUS population (ca. 1/500 kbp; Szurman-Zubrzycka et al., 2018), we can assume that each M_2_ plant carries more than 10,000 mutations. However, vast majority of them occur in non-coding regions, since genes (annotated coding sequences) make up 1.3% of barley genome (65.3 Mbp; Mascher et al., 2017). So, statistically, the number of mutations in genes equals to ca. 130 and, probably, most of them are silent and/or do not affect the protein function. Therefore, the probability of the presence of other deleterious mutation in a gene related to the same process of interest is very low. Nevertheless, in order to further decrease this probability, we performed backcrosses of the identified mutants with their parent variety ‘Sebastian’, which reduced the number of (putative) background mutations by half. The homozygous mutants selected from the F_2_ populations were phenotyped in this study. What is more, we observed that two different *dmc1.c* mutant lines (*dmc1.c-3041* and *dmc1.c-3223*), that originated from different M_1_ plants and possess different mutational background, show similar defects during meiotic divisions. These two lines share only the mutation leading to the R183K substitution in DMC1, whereas any putative background mutations differ between them, which strongly suggests that the identified mutation is responsible for this phenotype.

## Conclusion

The role of *DMC1* has been validated mostly in model plant species, such as *Arabidopsis thaliana* and rice. Here, we described functional analysis of *HvDMC1* in barley, which belongs to the most important cereal species worldwide. Our barley TILLING population, *Hor*TILLUS, has been used for mutational screening in the *HvDMC1* gene. We have identified and characterized a new allele, named *dmc1.c*, responsible for abnormalities during meiosis. Two mutated lines, from different M_1_ plants carrying the same mutation (G2571A that causes the R183K substitution), showed similar defects in this process, which strongly suggests that *HvDMC1* is involved in the proper course of meiosis in barley. We conclude that DMC1 is required for DSB repair during meiosis, the process which has yet to be fully elucidated.

## Data Availability

All datasets for this study are included in the manuscript and the Supplementary Files.

## Author Contributions

IS and DG conceived the project and designed the experiments. DG supervised the project. MS-Z, BB, MS-J, and JK conducted the research. MS-Z, IS, and DG wrote the manuscript.

## Conflict of Interest Statement

The authors declare that the research was conducted in the absence of any commercial or financial relationships that could be construed as a potential conflict of interest.

## Footnotes

1 www.ebi.ac.uk/Tools/msa/clustalo/

2 http://pfam.sanger.ac.uk/

3 http://plants.ensembl.org/index.html

4 http://webblast.ipk-gatersleben.de/barley_ibsc/viroblast.php
