# Supplementary material for: The dmc1 Mutant Allows an Insight Into the DNA Double-Strand Break Repair During Meiosis in Barley (Hordeum vulgare L.)
Source: Front Plant Sci. 2019 Jun 11;10:761. doi: 10.3389/fpls.2019.00761 (PMC6579892; doi:10.3389/fpls.2019.00761)

**Supplementary Material 1.**

Search for putative *HvDMC1* paralog(s) with the use of the IPK Barley BLAST Server (https://webblast.ipk-gatersleben.de/barley_ibsc/). *HvDMC1* gene sequence from NCBI (acc. no. AF234170.1) was used as a query sequence. The query was BLAST-ed against the Barley Genomic HC Gene database. The obtained result shows only one hit with high score that is the analyzed *HvDMC1* gene itself, with accession number HORVU5Hr1G040730, located on chromosome 5. No paralogs within the barley genome were identified.


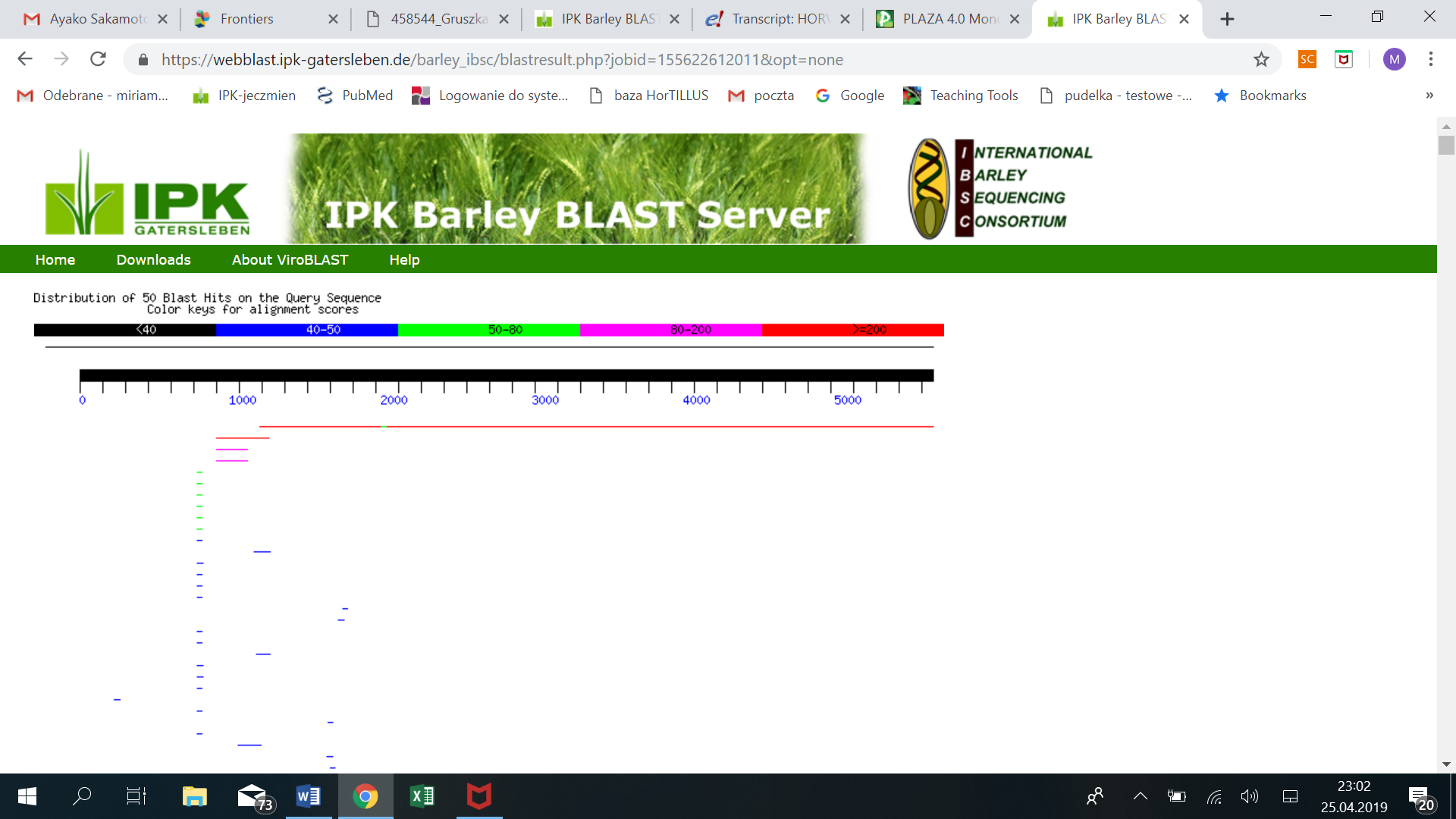


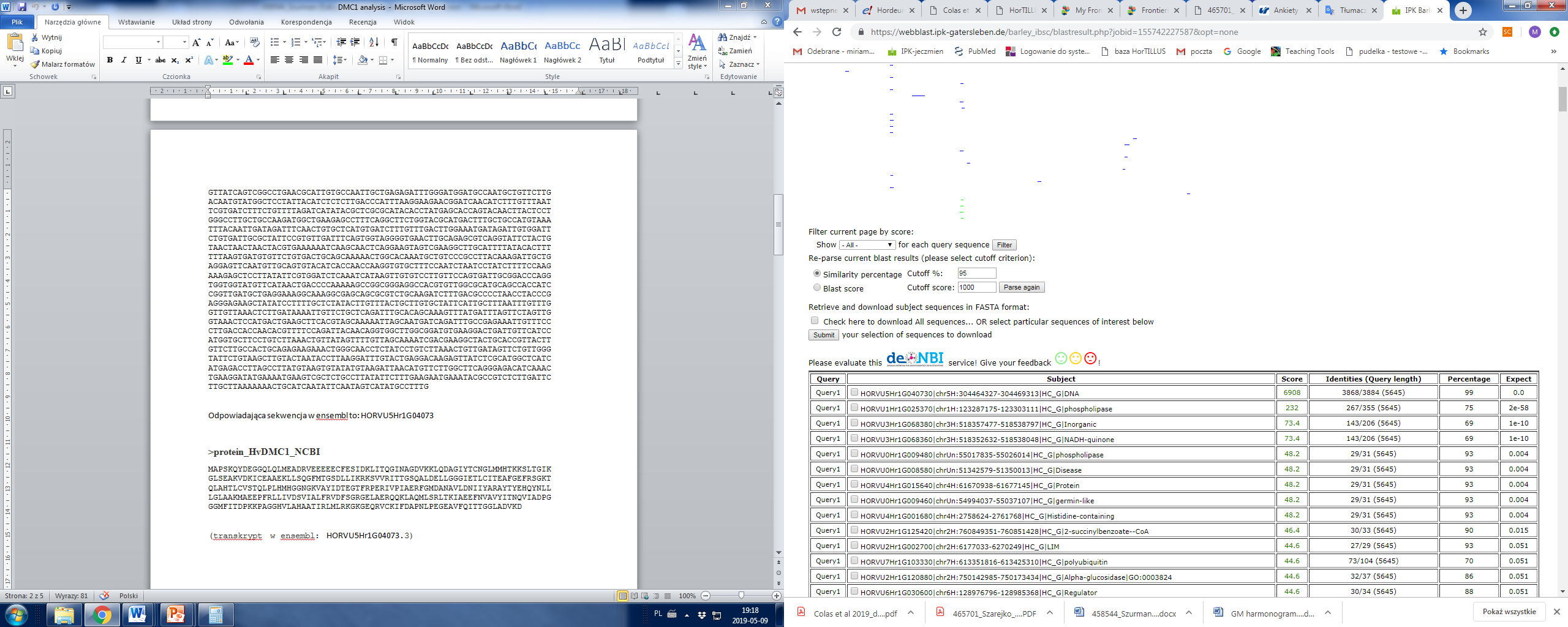

Supplement: Supplementary file 1 [file Table_1.DOCX]
