# Supplementary material for: The dmc1 Mutant Allows an Insight Into the DNA Double-Strand Break Repair During Meiosis in Barley (Hordeum vulgare L.)
Source: Front Plant Sci. 2019 Jun 11;10:761. doi: 10.3389/fpls.2019.00761 (PMC6579892; doi:10.3389/fpls.2019.00761)

**Supplementary Material** **2**.

The part of a phylogenetic tree created with the use of the PLAZA 4.0 Monocots tool. The section of the *Poaceae* family shows no barley paralogs. The HVU0041G0891 Plaza accession number (underlined with red color) corresponds to the HORVU5Hr1G040730 Ensembl accession number which represents the analyzed *HvDMC1* gene.


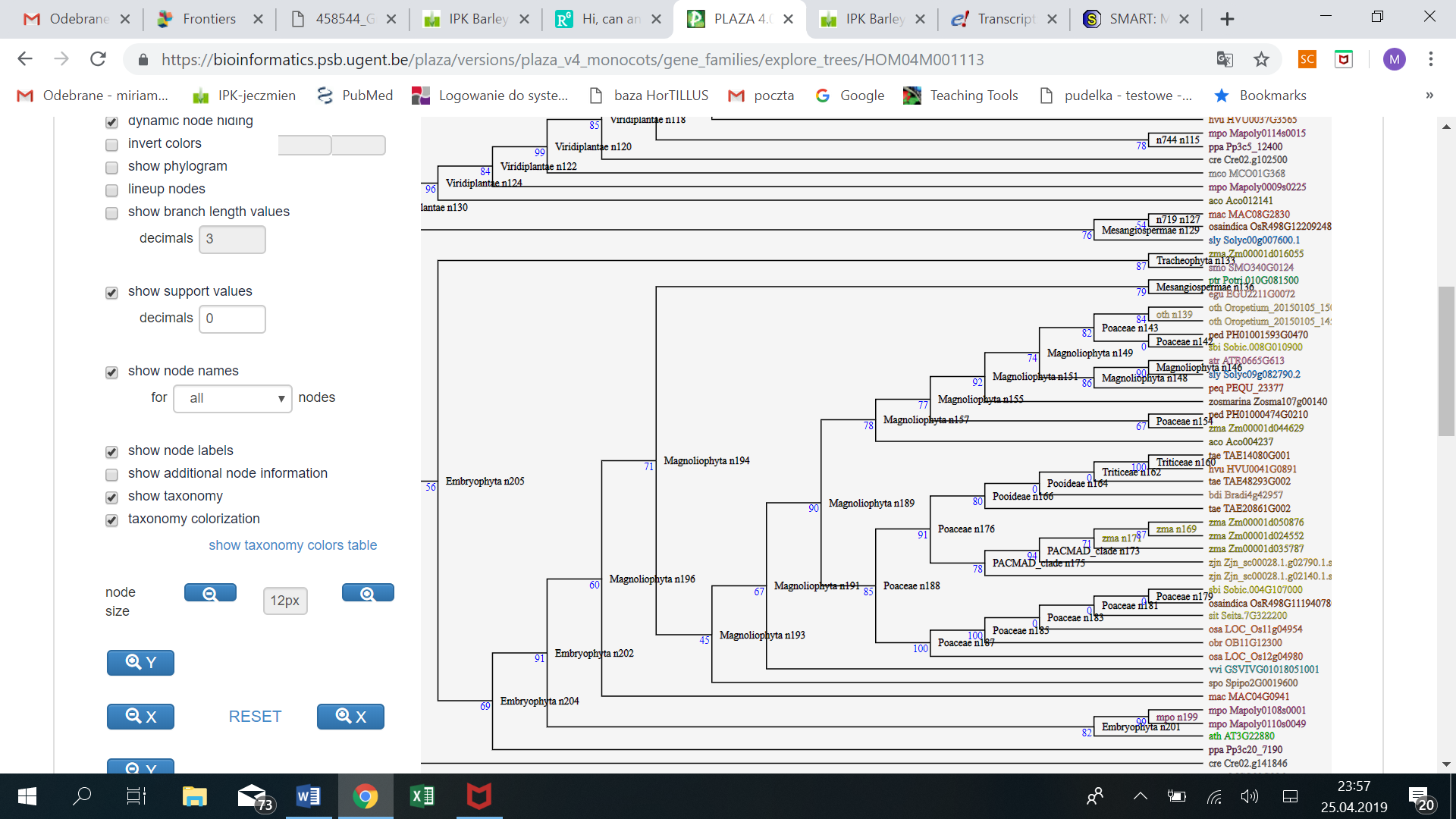

Supplement: Supplementary file 2 [file Table_2.DOCX]
