# Supplementary material for: The dmc1 Mutant Allows an Insight Into the DNA Double-Strand Break Repair During Meiosis in Barley (Hordeum vulgare L.)
Source: Front Plant Sci. 2019 Jun 11;10:761. doi: 10.3389/fpls.2019.00761 (PMC6579892; doi:10.3389/fpls.2019.00761)
Supplement: Supplementary file 3 [file Table_3.DOCX]

**Supplementary Material 3.**

The sequence of primers used for TILLING screening of the *HvDMC1* gene fragment:

HvDMC1.F: 5’- AGGTCAGGGAAGACCCAGTT-3’

HvDMC1.R: 5’- TGCTGCAGTCACAGAACACA-3’

Optimized PCR mix:

| - ddH_2_O | - 13,5 μl |
| --- | --- |
| - Buffer B for ColorTaq polymerase (Eurx) | - 2,0 μl |
| - dNTPs (5 mM) (Promega) | - 1,0 μl |
| - Primer HvDMC1.F* (10 pmol/μl) | - 0,5 μl |
| - Primer HvDMC1.R** (10 pmol/μl) | - 0,5 μl |
| - ColorTaq polymerase (Eurx) | - 0,5 μl |
| - DNA (100ng/µl) | - 2,0 μl |

*mixture of IRDye-700 labeled and unlabeled primers in 3:2 ratio

** mixture of IRDye-800 labeled and unlabeled primers in 4:1 ratio

Temperature profile of PCR reaction:

| - 1. Initial denaturation | - 94ºC | - 5 min |
| --- | --- | --- |
| - 2. Denaturation | - 94ºC | - 45 sec   x2 |
| - 3. Annealing | - 71ºC | - 45 sec |
| - 4. Elongation | - 72ºC | - 1 min |
| - 5. Denaturation | - 94ºC | - 45 sec   x2 |
| - 6. Annealing | - 70ºC | - 45 sec |
| - 7. Elongation | - 72ºC | - 1 min |
| - 8. Denaturation | - 94ºC | - 45 sec |
| - 9. Annealing | - 69ºC | - 45 sec |
| - 10. Elongation | - 72ºC | - 1 min |
| - 11. Denaturation | - 94ºC | - 45 sec |
| - 12. Annealing | - 68ºC | - 45 sec |
| - 13. Elongation | - 72ºC | - 1 min |
| - 14. Denaturation | - 94ºC | - 45 sec |
| - 15. Annealing | - 67ºC | - 45 sec |
| - 16. Elongation | - 72ºC | - 1 min |
| - 17. Denaturation | - 94ºC | - 45 sec |
| - 18. Annealing | - 66ºC | - 45 sec |
| - 19. Elongation | - 72ºC | - 1 min |
| - 20. Denaturation | - 94ºC | - 45 sec   x29 |
| - 21. Annealing | - 65ºC | - 45 sec |
| - 22. Elongation | - 72ºC | - 1 min |
| - 23. Final elongation | - 72ºC | - 3 min |
| - 24. Pause | - 8ºC | - ∞ |
