# Supplementary material for: The dmc1 Mutant Allows an Insight Into the DNA Double-Strand Break Repair During Meiosis in Barley (Hordeum vulgare L.)
Source: Front Plant Sci. 2019 Jun 11;10:761. doi: 10.3389/fpls.2019.00761 (PMC6579892; doi:10.3389/fpls.2019.00761)
Supplement: Supplementary file 4 [file Table_4.DOCX]

**Supplementary Material 4.**

The images of polyacrylamide gels (from 700 nm and 800 nm LI-COR channels) showing results of TILLING for the *HvDMC1* amplicon. The full PCR products (811 bp) are visible in both channels. The additional fragments that are seen in two channels at different positions (in red frames), arise after the cleavage of mismatches in heteroduplexes (their lengths sum up to total length of the PCR product). These additional bands indicate bulks with potential mutations. All potential mutations were confirmed by sequencing.


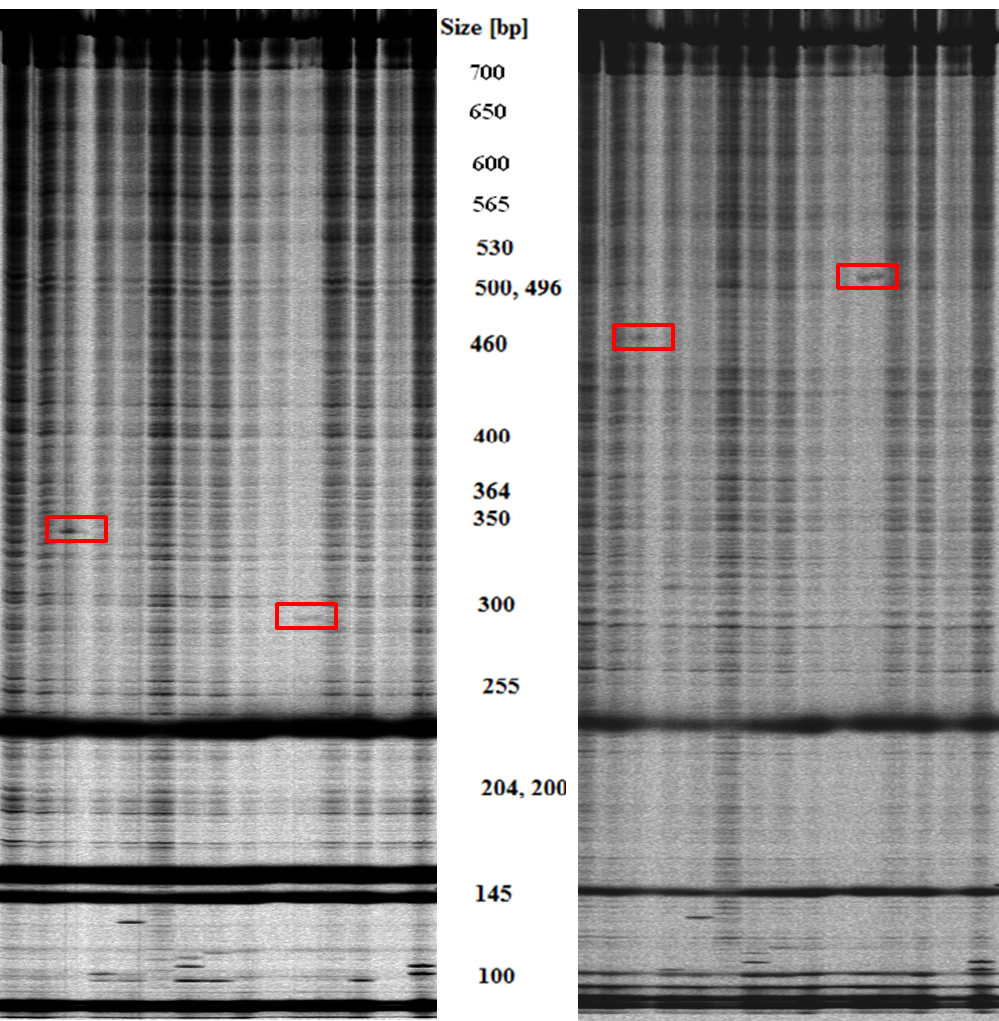


800 nm channel

700 nm channel
